# Low grade Endometrial Stromal Sarcoma of uterine corpus, a clinico-pathological and survey study in 14 cases

**DOI:** 10.1186/1477-7819-4-50

**Published:** 2006-08-09

**Authors:** Tahereh Ashraf-Ganjoei, Nadereh Behtash, Mamak Shariat, Asamosadat Mosavi

**Affiliations:** 1 Gynecologic Oncology Department, Vali Asr Hospital, Keshavarz Blvd., Tehran 14194, Iran

## Abstract

**Background:**

Endometrial stromal sarcoma (ESS) is a rare disease with probably less than 700 new cases in the USA or Europe per year. The aim of this study was to evaluate the behavior of low-grade endometrial stromal sarcoma (LGESS) in relation to their clinical and pathological features and to identify possible prognostic factors.

**Patients and methods:**

Fourteen patients with histologically proven ESS were included in the analysis. Endometrial stromal sarcoma is characterized by proliferations composed of cells with Endometrial stromal cell differentiation. Low-grade endometrial stromal sarcoma has an infiltrating margin and typically show extensive worm-like vessel invasion.

**Results:**

The median age was 44.35 ± 6 years. The most common presenting symptom was vaginal bleeding, occurring in twelve patients (86%). Diagnosis was made through Fractional dilatation and curettage in four patients (28.5%). Eight patients had a total abdominal hysterectomy and salpingo-ophorectomy (57%). Radiotherapy as adjuvant therapy was administered to four patients (28.5%). The median follow-up time was 45.6 months (range 24–84). The median overall survival of the 14 patients was 45.35 ± 21 months (range 20–83). Three of 14 patients demonstrated a recurrence of disease at 9, 72, and 96 months respectively. The recurrent diseases were treated with surgery, chemotherapy, and radiotherapy. No patient died of the disease. Clinico-pathological parameters did not significantly differ between patients with and without recurrence, but patients with no myometrial invasion and low mitotic count <= 5/HPF showed longer disease-free survival.

**Conclusion:**

Five-year survival rate was 93%. Survival probabilities were calculated by the product limit method of Kaplan and Meier that showed, patients with no myometrial invasion and low mitotic count <= 5/HPF have longer disease-free survival, but P value was not significant.

## Background

Endometrial Stromal Sarcomas (ESSs) are very rare malignant tumors that constitute approximately 10% of all uterine sarcomas but only around 0.2% of all uterine malignancies [1]. The annual incidence of ESS is 1–2 per million women accounting for 400 to 700 new cases each year in Europe [2].

ESS can be mistaken for leiomyoma. Its clinical recognition may be difficult, and the diagnosis is often made postoperatively after histological examination [3,4]. The typical gross appearances of ESS are a single nodule, multiple solid-cystic masses, and a poorly demarcated lesion with occasional cystic degeneration or rarely cystic multilocular lesion [5]. There are three types of endometrial stromal tumors: endometrial stromal nodule, low-grade ESS (LGESS), and high-grade ESS (HGESS). Only the nature of the margin, histologically differentiate LGESS from stromal nodule. The division of endometrial stromal sarcomas into low-grade and high-grade categories has fallen out of favor, and the term endometrial stromal sarcomas now considered best restricted to neoplasms that were formally to as low-grade endometrial stromal sarcomas [6]. High-grade tumors without recognizable evidence of a definite endometrial stromal phenotype are now termed endometrial sarcomas [7].

The pathogenesis of these lesions remains unknown, but exposure to tamoxifen and unopposed estrogens has been implicated in some cases [8].

Uterine sarcomas most often affect postmenopausal women [9]. Women with LGESS are younger than women with other uterine sarcomas, with a median age between 45 and 57 years and, generally do not have the usual risk factors for endometrial cancer. Symptoms at presentation include abnormal vaginal bleeding, progressive menorrhagia, and abdominal pain. While often indolent in behavior, ESS is malignant, and up to 30% of women with low-grade ESS have extra uterine disease at presentation.

Surgery is fundamental in LGESS as other sarcoma. Management and treatment generally consists of total abdominal hysterectomy and bilateral salpingo-oophorectomy. Due to the high recurrence risk even with localized tumors, many clinicians advocate use of adjuvant chemotherapy, radiation therapy, and/or hormone therapy to suppress tumor growth [4]. There is no firm evidence from a prospective study that adjuvant chemotherapy or radiation therapy is of substantial benefit for patients with uterine sarcoma. Postoperative pelvic radiotherapy reduces local recurrence but has not been consistently shown to prolong survival.

Progestin therapy has been reported to reduce the risk of recurrence when used in the adjuvant setting. Most women with LGESS undergo bilateral salpingo-oophorectomy as part of primary treatment but estrogen also can be produced by extraovarian sources.

These tumors typically have an indolent growth with a tendency for late recurrence [6]. Pelvic or abdominal recurrences in stage I disease develop in one-third to one-half of patients [10].

The aim of this study was to evaluate the clinical outcome and the behavior of LGESS in relation to its clinical and pathological features.

## Patients and methods

This study includes 14 patients with histologically proven low-grade endometrial stromal sarcoma treated at of Gynecologic Oncology Department of the Vali-Asr University Hospital, Tehran, Iran, between 1999 to 2005. Hospital records and available histological material for each patient were reviewed retrospectively. Slides from each patient were reexamined to confirm the diagnosis.

Patients with a diagnosis of high-grade endometrial stromal sarcoma were not included. Demographic information, pathologic, and treatment information were collected from the clinic and hospital charts. All had primary surgical management. Eight patients underwent total abdominal hysterectomy and salpingo-oophorectomy, and three patients underwent subtotal hysterectomy and salpingo-oophorectomy. In one patient, subtotal hysterectomy without salpingo-oophorectomy was performed. They had regular follow-up visits until the end of study.

The end point of 5-year survival was used for analysis. Survival probabilities were calculated by Kaplan and Meier method. Correlation between recurrence and clinicopathological parameters (myometrial invasion, mitotic count) was tested using the Fisher Exact test. P values of less than 0.05 were considered statistically significant.

## Results

Fourteen patients with LGESS were identified at our institution from 1999 to 2005. The median age was 44.35 ± 6 years (range 33–52). The mean parity of the patients was 4.4 (range 0–8). The most common presenting symptom was vaginal bleeding, occurring in twelve patients(86%) while other features such as pelvic mass and acute abdominal pain were observed in 1 (7%) and 1 (7%) patients, respectively (table 1). Clinical impression in four patients (29%) was uterine myoma.

**Table 1:** patient characteristics.

No	Age	Parity	Symptom	D&C	Type of Operation	Myometrial Invasion	Mytotic Count	Irradiation	Recurrence
**1**	49	2	AUB	Yes	TAH&BSO	Low	=<5/10HPF	Yes	
**2**	42	6	AUB	Yes	TAH&BSO	Deep	>5/10HPF	Yes	
**3**	51	3	AUB	No	TAH&RSO	No	=<5/10HPF	Yes	
**4**	52	5	AUB	No	TAH&RSO		>5/10HPF	No	
**5**	38	7	AUB	Yes					
**6**	37	3	Pelvic Mass	No	Trachelectomy & RSO	No	=<5/10HPF	Yes	Yes
**7**	48	8	AUB	No	TAH			No	Yes
**8**	33	5	AUB	No	TAH&BSO	No	>5/10HPF	No	Yes
**9**	44	Virgin	AUB	No	TAH&BSO	No			
**10**	48	3	AUB	No	subTAH&BSO	No	=<5/10HPF	No	
**11**	38	4	Acute Abdomen	No	subTAH	No			
**12**	50	1	AUB	No	subTAH&BSO	No	=<5/10HPF	No	
**13**	48	8	AUB	Yes	TAH&BSO	Low	=<5/10HPF	No	
**14**	43	7	AUB	No	subTAH&BSO	No	=<5/10HPF	No	

AUB: Abnormal uterine bleeding; TAH: total abdominal hysterectomy; HPF: high power field; BSO: Bilateral salpingooopherectomy; RSO: Residual salpingooopherectomy

Fractional dilatation and curettage (FD&C) was performed in four patients presenting with abnormal uterine bleeding (AUB). Diagnosis was made through FD&C in four patients. The other 10 patients were diagnosed by hysterectomy. All the histological slides were reexamined by the same pathologist.

Eight patients had a total abdominal hysterectomy and four patients had a subtotal hysterectomy as part of their initial surgical treatment. One patient underwent trachelectomy following the previous subtotal hysterectomy. Radiotherapy as adjuvant therapy was administered to four patients (29%).

The median follow-up time was 45.6 months (range 24–84). Five-year survival rate was 93%. The median overall survival of the 14 patients was 45.35 ± 21 months (range 20–83). Only one patient recurred at 9 months. Two of 14 patients demonstrated a recurrence of disease at 6 years, and 8 years respectively (table 2). Recurrent disease in pelvic was treated by combination chemotherapy and surgery in one patient. The second patient with recurrence in pelvic and lung received combination chemotherapy and external radiotherapy. In the third patient, recurrence in vagina and lung was treated with combination chemotherapy only. No patient died of the disease. Twelve patients are alive without disease and two alive with disease.

**Table 2:** Recurrent Cases

No of patients	**Disease-Free survival**	**Sites of recurrence**	**Treatment of Recurrence**		
			
			**Chemotherapy**	**Irradiation**	**surgery**
6	9 months	Pelvic and lung	Yes(first)	Yes	No
7	8 years	Vagina and lung	Yes	No	No
8	6 years	pelvic	Yes	No	Yes(first)
					

**Figure 1:**
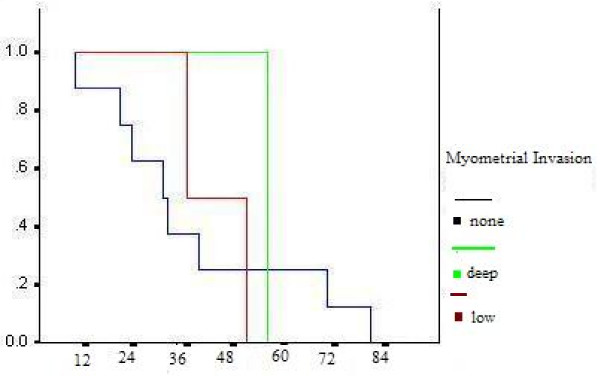
Survival of patients with LGESS based on myometrial invasion.

**Figure 2:**
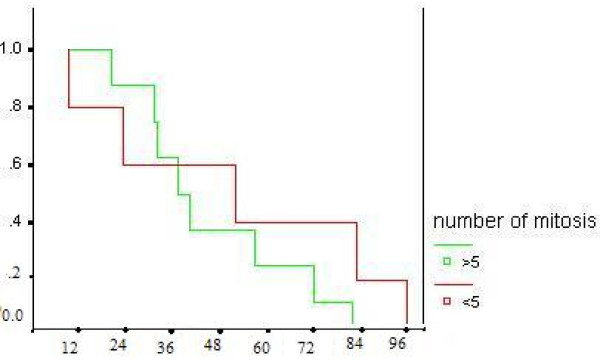
Survival of patients with LGESS based on number of mitosis.

Five-year survival rate was93%. Survival probabilities were calculated by the product limit method of Kaplan and Meier that showed, patients with no myometrial invasion and low mitotic count <= 5/HPF have longer disease-free survival (fig 1 and fig 2), but P value was not significant.

## Discussion

ESS are very rare malignant tumors that make up approximately 10% of all uterine sarcomas but only around 0.2% of all uterine malignancies [11].

ESS are divided in low and high-grade tumors according to cell morphology and mitotic count [6], Boardman CH, et al defined low-grade ESS from high-grade ESS by the cellular uniformity, less frequent mitosis (<3 per 10 high-power fields versus >10), and lack of hemorrhage and necrosis [3]. However, there are controversies surrounding the separation of endometrial stromal sarcomas into high and low grade based on mitotic activity [12]and at present, mitotic counts are no longer used to differentiated high-grade from low-grade lesions [7]. Accordingly, several authors have concluded that two separate disease entities exist and, respectively, that HGESS should be regarded as an undifferentiated sarcoma or as a unique type of high-grade uterine sarcoma (e.g. carcinosarcoma without any detectable carcinoma portion) [13].

Low-grade endometrial stromal sarcoma (LGESS) has an infiltrating margin and typically shows extensive worm-like vessel invasion [6].

Most patients are in the age range of 42 to 53 years. More than half the patients are premenopausal. Young women and girls may be affected. In this study, 79% of patients were premenopausal.

Abnormal vaginal bleeding is the most common presenting symptom, and abdominal pain and uterine enlargement may occur [9,7]. We showed abnormal vaginal bleeding in 86% of the patients. Clinical impression in four cases was uterine myoma (28.5%) and one patient had presented with severe abdominal pain.

The median follow-up time was 45.6 months (range 24–84). Five-year survival rate was 93%. In this study, the median overall survival of the 14 patients was 45.35 ± 21 months (range 20–83).

Although the bulk of the tumor is almost always intramyometrial [14], most endometrial stromal sarcomas involve the endometrium, and uterine curettage usually leads to diagnosis [7]. In our study, the diagnosis in four patients (28.5%) was made through D&C. The other 10 patients were diagnosed by hysterectomy (71%). Surgery has always been described as the most effective treatment in LGESS as other uterine sarcomas. Primary surgery was performed in 13 patients in our series. The one patient refused from any treatment such as surgery.

The efficacy of adjuvant therapy in patients with ESS is still not proven [15]. In our study radiotherapy as adjuvant, therapy was administered to four patients (28.5%), but one of them recurred at 9 months.

Although LGESS behavior is relatively indolent, late recurrence and distant metastases may occur [14]. The risk of recurrence is thought to be as high as 50%, although these tumors are usually slow growing and recurrences occur late. In one large series, the interval before recurrence varied from 3 months to 23 years, with a median interval of 3 years. In the largest clinico-pathologic study to date on ESS, the median time between hysterectomy and relapse was 5.4 years and 9 month for stages 1 and 3–4, respectively [16]. We showed only one recurrence at 9 months, but two recurrences occurred at 6 and 8 years respectively.

In Brunisholza study, the recurrent disease mainly spread to the pelvis, lower genital tract, lungs, and rarely to other site [17]. Recurrence sites in our patients were vagina, pelvis, and lung.

Prolonged survival and even cure are common after surgical resection of recurrent or matastatic lesions [7]. One of our patients is alive 3 years without disease after resection of vaginal metastasis and chemotherapy.

Uterine sarcomas have a poor prognosis, and survival is much worse than that reported for endometrial adenocarcinoma, with an overall survival of less than 50% at 2 years, even when presenting at an early stage [18]. A higher survival probability for patients with LGESS compared to other uterine sarcoma is often reported [19]. In this study, five-year survival rate was 93%.

Prognostic factors in patients with ESS are still discussed controversially [11]. The negative prognostic influence of a high mitotic count was revealed in previous studies [20]. In the present study, survival probabilities were calculated by the product limit method of Kaplan and Meier that showed, patients with no myometrial invasion and low mitotic count <= 5/HPF have longer disease-free survival, but P value was not significant.

## Conclusion

In summary, we found not significant association between mitotic count, myometrial invasion and risk of recurrence. The large variation in pathologic characteristics and in treatment policies, combined with the scarcity of patients, has meant that in the past, there has been a general feeling that there is insufficient information on optimal managements and their influence on tumor behavior. The difficulty in obtaining information on tumor behavior and best treatment has led different authors to study of the prognostic factors. Additional studies on a larger group of patients allowing multi analysis are necessary to be better able to predict the prognosis of patients with ESS and to define the exact role of adjuvant therapy.

## Competing interests

The author(s) declare that they have no competing interest

## Authors' contributions

**TAG **participated in the design of the study and participated in drafting the manuscript.

**NB **carried out the surgery and helped to draft the manuscript.

**MS **participated in drafting the manuscript.

**AM **carried out the chemotherapy and follow-ups.

All authors read and approved the final manuscript.
